# An experimental study of sexual function improving effect of *Myristica fragrans *Houtt. (nutmeg)

**DOI:** 10.1186/1472-6882-5-16

**Published:** 2005-07-20

**Authors:** Tajuddin, Shamshad Ahmad, Abdul Latif, Iqbal Ahmad Qasmi, Kunwar Mohammad Yusuf Amin

**Affiliations:** 1 Department of Ilmul Advia (Unani Pharmacology), Faculty of Unani Medicine, Aligarh Muslim University, Aligarh-202002, India

## Abstract

**Background:**

*Myristica fragrans *Houtt. (nutmeg) has been mentioned in Unani medicine to be of value in the management of male sexual disorders. The present study was undertaken to evaluate the aphrodisiac effect of 50% ethanolic extract of nutmeg along with its likely adverse effects and acute toxicity using various animal models.

**Methods:**

The suspension of the extract was administered (100, 250 and 500 mg/kg, p.o.) to different groups of male rats daily for seven days. The female rats involved in mating were made receptive by hormonal treatment. The general mating behaviour, libido and potency were studied and compared with the standard reference drug sildenafil citrate. Likely adverse effects and acute toxicity of the extract were also evaluated.

**Results:**

Oral administration of the extract at the dose of 500 mg/kg, produced significant augmentation of sexual activity in male rats. It significantly increased the Mounting Frequency, Intromission Frequency, Intromission Latency and caused significant reduction in the Mounting Latency and Post Ejaculatory Interval. It also significantly increased Mounting Frequency with penile anaesthetisation as well as Erections, Quick Flips, Long Flips and the aggregate of penile reflexes with penile stimulation. The extract was also observed to be devoid of any adverse effects and acute toxicity.

**Conclusion:**

The resultant significant and sustained increase in the sexual activity of normal male rats without any conspicuous adverse effects indicates that the 50% ethanolic extract of nutmeg possesses aphrodisiac activity, increasing both libido and potency, which might be attributed to its nervous stimulating property. The present study thus provides a scientific rationale for the traditional use of nutmeg in the management of male sexual disorders.

## Background

Nutmeg is the dried kernel of broadly ovoid seed of *Myristica fragrans *Houtt. (Myristicaceae), a bushy evergreen tree 10–20 m high, indigenous to India, Indonesia and Srilanka [1,2]. It is now cultivated in many tropical countries of both the hemispheres [3]. *M. fragrans *commonly known as nutmeg is widely used as spice and in alternative medicine it has been reported to have aphrodisiac [4,5], stomachic, carminative [6], tonic [7], nervous stimulant [8], aromatic, narcotic, astringent, hypolipidemic, antithrombotic, antifungal, antidysentric and anti inflammatory properties [9]. It is reported to be useful in paralysis [10] and increases blood circulation [11]. It has also been reported to have antioxidant property [12]. Phytochemical studies indicate that nutmeg contains a volatile oil, a fixed oil, proteins, fats, starch and mucilage. The fixed oil contains myristin and myristic acid. Nutmeg yields 5–15% of volatile oil, which contains pinene, sabinene, camphene, myristicin, elemicin, isoelemicin, eugenol, isoeugenol, methoxyeugenol, safrole, dimeric phenylpropanoids, lignas and neolignas [1,13,14] and by expression it yields a product known as nutmeg butter. Eugenol the major constituent inhibits lipid peroxidation and maintains activities of enzymes like superoxide dismutase, catalase, glutathione peroxidase, glutamine transferase and glucose-6-phosphate dehydrogenase [15], and has also been reported to have vasodilatory [16] and smooth muscle relaxant property [17]. There is no information regarding the chemical constituents in 50% ethanolic extract of nutmeg, but their amount in the extract seems to be lower than in nutmeg. However, its preliminary phytochemical studies that were performed as described by Jenkins et al. [18] indicate the presence of sterols, phenols, alkaloids and. Amino acids. The chloroform extract of nutmeg showed analgesic and anti-inflammatory activity in rodents [19], and also exhibited antidiarrheal activity by increasing tissue contents of Na^+ ^and Cl^- ^ions [20]. The petroleum ether extract of *M. fragrans *fruits possesses anti-diarrheal property [21], and its n-hexane extract has been reported to have memory enhancing effect in mice [22]. The ethanolic extract (50%) of nutmeg exhibited significant aphrodisiac effect in male mice in our earlier study, in which only mounting frequency and mating performance were considered as the markers for sexual function [23].

Considering above information, nutmeg-extract (50% ethanolic) was subjected to a detailed screening for sexual function improving activity using various animal models. The standard drug sildenafil citrate was used as a referent for quantitative comparison. Likely treatment-related adverse effects and acute toxicity were also evaluated. The doses used in the study were selected according to Freirich[24], multiplying the Unani clinical doses [10,25,26] by the conversion factor of 7.

## Methods

### Plant material

Dried kernel of *M. fragrans *(nutmeg) was procured from the market (Delhi, India). The plant material was authenticated by Prof. S. H. Afaq, In-charge, Pharmacognosy Section, Department of Ilmul Advia, Faculty of Unani Medicine, Aligarh Muslim University, Aligarh, India. A voucher (M144) sample was deposited for further reference.

### Extraction procedure

Nutmeg was crushed to coarse powder and sieved through No. 20–40 mesh sieve and refluxed by mixing the powdered nutmeg with 1:3 w/v in 50% ethanol v/v by Soxhlet apparatus for 6 h. The extract was filtred and the solvent from the filtrate was removed by rotary evaporator under reduced pressure and low temperature. The yield of extract was 21.20% w/w in terms of dried starting material. The extract was preserved in a refrigerator.

### Chemicals

Sildenafil citrate was obtained from Zydus Cadila (Ahmadabad, India). Ethinyl oestradiol was a gift from Infar Limited (Kolkata, India). Progestereon was procured from Sun Pharmaceutical Industries Limited (Mumbai, India) and 5% xylocaine ointment was obtained from Astra IDL Limited (Bangalore, India).

### Animals

Three month old male and female albino rats of Wistar strain weighing 350–400 g and 225–275 g, respectively, were used for aphrodisiac study. Adult albino mice of either sex weighing 25–35 g were used for acute toxicity testing. All the animals were housed singly in separate standard propylene cages and maintained under standard laboratory conditions (temperature 24–28°C, relative humidity 60–70%, 12 h light-dark cycle) with water and food (Gold Mohar, Lipton-India) *ad libitum*. The Departmental ethical committee for animal care and use approved the experimental design.

### Drug preparation

Nutmeg in Unani medicine is orally administered, therefore the extract was suspended in distilled water using Tween-80 (1%) for oral administration. Similarly sildenafil citrate and ethinyl oestradiol were also suspended in distilled water using Tween -80 (1%) separately, for oral use. Progesterone was dissolved in olive oil for subcutaneous injection. All the drug solutions were prepared just before administration.

### Mating behaviour test

The effect of the test drug on mating behaviour was studied according to the methods described by Dewsbury and Davis Jr. [27] and Szechtman et al. [28], modified by us. Healthy and sexually experienced male rats were selected for the study. They were divided in to five groups each consisting of six rats and placed individually in separate propylene cages during the experiment. Group 1 served as control group and received 10 ml/kg of distilled water orally, daily for 7 days at 18:00 h. Groups 2–4 received suspension of the extract orally at the doses of 100, 250 and 500 mg/kg, respectively, once a day for 7 days at 18:00 h. Group 5 served as standard group and given suspension of the standard drug 1 h before the commencement of the experiment. Since the male animals should not be tested in unfamiliar circumstances the animals were brought to the laboratory and exposed to dim light (in 1 w fluorescent tube in a laboratory of 14' × 14') at the stipulated time of testing daily for 6 days before the experiment.

The female rats allow mating only during the estrus phase. Thus, they were artificially brought into oestrus (heat) by the method of Szechtman et al [28]. They were administered suspension of ethinyl oestradiol orally at the dose of 100 μg/animal 48 h prior to the pairing plus progesterone injected subcutaneously, at the dose of 1 mg/animal 6 h before the experiment. The receptivity of the female animals was confirmed before the test by exposing them to male animals, other than the control, test and standard animals. The most receptive females were selected for the study. The experiment was carried out on the 7^th ^day after commencement of the treatment of the male animals. The experiment was conducted at 20:00 h in the same laboratory and under the light of same intensity. The receptive female animals were introduced into the cages of male animals with 1 female to 1 male. The observation for mating behaviour was immediately commenced and continued for first 2 mating series. The test was terminated if the male failed to evince sexual interest. If the female did not show receptivity it was replaced by another artificially warmed female. The occurrence of events and phases of mating were called out to be recorded on an audio-cassette as soon as they appeared. Their disappearance was also called out and recorded. Later, the frequencies and phases were determined from cassette transcriptions: number of mounts before ejaculation or Mounting Frequency (MF), number of intromission before ejaculation or Intromission Frequency (IF), time from the introduction of female into the cage of the male upto the first mount or Mounting Latency (ML), time from the introduction of the female up to the first intromission by the male or Intromission Latency (IL), time from the first intromission of a series upto the ejaculation or Ejaculatory Latency (EL), and time from the first ejaculation upto the next intromission by the male or Post Ejaculatory Interval (PEI). In the second mating series only the EL was recorded. The values for the observed parameters of the control, test and standard animals were statistically analysed by using one-way analysis of variance (ANOVA) method.

### Test for libido

The test was carried out by the method of Davidson [29], modified by us. Sexually experienced male rats were divided into five groups each consisting of six rats and kept singly in separate propylene cages during the experiment. Group 1 represented the control group, which received 10 ml/kg of distilled water orally, once a day for 7 days at 18:00 h. Group 2–4 received suspension of the extract orally at the doses of 100, 250 and 500 mg/kg, respectively, daily for 7 days at 18:00 h. Group 5 served as standard group and given suspension of the standard drug orally at the dose of 5 mg/kg, 1 h prior to the commencement of the experiment. The female rats were made receptive by hormonal treatment and all the animals were accustomed to the testing condition as previously mentioned in mating behaviour test. The animals were observed for Mounting Frequency (MF) on the evening of 7^th ^day at 20:00 h. The penis was exposed by retracting the sheath and 5% xylocaine ointment was applied 30, 15 and 5 min before starting observations. Each animal was placed individually in a cage and the receptive female rat was placed in the same cage. The number of mountings was noted. The animals were also observed for intromission and ejaculation. The MF in control, test and standard animals was statistically analysed by employing one-way analysis of variance (ANOVA) method.

### Test for potency

The test was carried out by the methods of Hart and Haugen [30] and Hart [31], modified by us. The male rats were divided in to five groups each consisting of six rats and placed individually in separate propylene cages during the experiment. Group 1 represented the control group, which received 10 ml/kg of distilled water orally daily for 7 days. Group 2–4 received suspension of the test drug orally at the dose of 100, 250 and 500 mg/kg, respectively, daily for 7 days. Group 5 served as standard group and received suspension of the standard drug orally at the dose of 5 mg/kg, 1 h prior to the commencement of the experiment. On the 8^th ^day, the test for penile reflexes was carried out by placing the rat on its back, in a glass cylinder for partial restraint. The preputial sheath was pushed behind the glans by means of thumb and index finger and held in this manner for a period of 15 min. Such stimulation elicits a cluster of genital reflexes. The following components were recorded: Erections (E), Quick Flips (QF) and Long Flips (LF). The frequency of these parameters observed in control, test and standard groups was statistically analysed by using one-way analysis of variance (ANOVA) method.

### Adverse effects

All treated rats were observed at least once daily for any overt sign of toxicity (salivation, rhinorrhoea, lachrymation, ptosis, writhing, convulsions and tremors), stress (erection of fur and exophthalmia) and changes in behaviour (such as spontaneous movement in the cage, climbing, cleaning of face). In addition, food and water intake was noted.

### Acute toxicity testing

The acute toxicity of the extract was studied in adult albino mice of either sex. They were divided into five groups each consisting of six mice. The suspension of the extract was administered orally at four different doses of 500, 1000, 2000 and 4000 mg/kg, respectively, to different groups of mice separately. Control animals received 10 ml/kg of distilled water orally. The animals were observed continuously for the initial 4 h for behavioural changes and mortality and intermittently for the next 6 h and then again at 24 h and 48 h after dosing. The behaviour parameters observed were convulsion, hyperactivity, sedation, grooming, loss of righting reflex and increased respiration.

### Statistical analysis

The significance of difference between the means was determined by one-way analysis of variance (ANOVA) with post-hoc't' test. P value <0.05 was considered as significant.

## Results

### Effect of the extract on mating behaviour

The results of mating behaviour test show that the extract at the dose of 500 mg/kg, significantly increased the Mounting Frequency (MF) (P < 0.001), Intromission Frequency (IF) (P < 0.001), Ejaculatory Latency in first series (EL_1_) (P < 0.001), Ejaculatory Latency in second series (EL_2_) (P < 0.001), and caused significant reduction in the Mounting Latency (ML) (P < 0.001), Intromission Latency (IL) (P < 0.01) and Post Ejaculatory Interval (PEI) (P < 0.001), as compared to control group. The dose of 250 mg/kg of the extract significantly increased the MF(P < 0.001), IF (P < 0.01) and significantly decreased the ML (P < 0.01), PEI (P < 0.001), IL (P < 0.05), EL_1 _(P < 0.05) and did not significantly alter the EL_2, _in comparison with the control group. Whereas, the extract at the dose of 100 mg/kg, significantly increased the MF (P < 0.01) and PEI(P < 0.05), but did not affect the IF, ML, IL, EL_1 _and EL_2 _in a significant manner as compared to control group. However, the standard drug increased the MF (P < 0.001), IF (P < 0.001), EL_1 _(P < 0.001), EL_2 _(P < 0.001) and PEI (P < 0.001) as well as decreased the ML (P < 0.001) and IL (P < 0.001) in a highly significant manner as compared to control animals (Table 1).

**Table 1:** Effect of 50% ethanolic extract of *M. fragrans *(nutmeg) on mating behaviour in male rats

Parameters	Mean ± SEM				
	
	Control (10 ml/kg)	Nutmeg (100 mg/kg)	Nutmeg (250 mg/kg)	Nutmeg (500 mg/kg)	Sildenafil citrate (5 mg/kg)
Mounting Frequency (MF)	11.50 ± 1.22	14.3 ± 0.49**	23.70 ± 1.47***	43.80 ± 0.94***	48.70 ± 2.34***
Intromission Frequency (IF)	5.50 ± 1.22	5.50 ± 0.42 NS	8.17 ± 0.60**	12.50 ± 0.76***	24.70 ± 0.81***
Mounting Latency (ML, in sec)	35.30 ± 1.51	35.80 ± 1.46 NS	28.50 ± 0.34**	22.80 ± 0.87***	11.70 ± 1.37***
Intromission Latency (IL, in sec)	40.00 ± 5.29	37.70 ± 1.97 NS	34.00 ± 1.65*	27.50 ± 1.80**	15.00 ± 0.89***
Ejaculatory Latency in first series (EL_1_, in sec)	198.00 ± 0.98	211.00 ± 6.69 NS	218.00 ± 2.13*	235.00 ± 6.65***	344.50 ± 12.00***
Ejaculatory Latency in second series (EL_2_, in sec)	297.33 ± 8.10	300.00 ± 4.98 NS	319.00 ± 6.98 NS	358.00 ± 7.22***	398.16 ± 13.50***
Post Ejaculatory Interval (PEI, in sec)	364.00 ± 12.22	336.00 ± 7.92*	301.00 ± 6.89***	224.00 ± 4.69***	99.00 ± 5.68***

Tabular values are mean ± SEM, n = 6 (number of animals in each group); significant difference from control, NS: Not significant. *P < 0.05, **P < 0.01; ***P < 0.001.

### Effect of the extract on libido

The results obtained with the test for libido show that the extract at the dose of 500,250 and 100 mg/kg, significantly increased the Mounting Frequency (MF) (P < 0.001, P < 0.01 and P < 0.05, respectively) as compared to control group. The standard drug also significantly increased the MF (P < 0.001) as compared to control animals. Intromission and Ejaculation were absent in control, test and standard groups (Table 2).

**Table 2:** Effect of 50% ethanolic extract of *M. fragrans *(nutmeg) on mounting frequency (test for libido) in male rats

Parameters	Mean Frequency ± SEM				
	
	Control (10 ml/kg)	Nutmeg (100 mg/kg)	Nutmeg (250 mg/kg)	Nutmeg (500 mg/kg)	Sildenafil citrate (5 mg/kg)
Mounting Frequency (MF)	6.17 ± 0.98	7.83 ± 0.47*	8.50 ± 0.56**	14.50 ± 4.43***	23.00 ± 2.17***
Intromission Frequency (IF)	Nil	Nil	Nil	Nil	Nil
Ejaculation (EJ)	Absent	Absent	Absent	Absent	Absent

Tabular values are mean ± SEM, n = 6 (number of animals in each group); significant difference from control, *P < 0.05, **P < 0.01; ***P < 0.001.

### Effect of the extract on potency

The test for potency revealed that the extract at the dose of 500 mg/kg, significantly increased the frequency of Erections (E) (P < 0.001), Quick Flips (QF) (P < 0.001) and Long Flips (LF) (P < 0.001) as well as the aggregate of these penile reflexes (TPR) (P < 0.001) in comparison with the control group. The test drug at the dose of 250 mg/kg, significantly increased the E (P < 0.05), LF (P < 0.01) and TPR (P < 0.05) but did not significantly affect the QF. Whereas, the extract at the dose of 100 mg/kg, did not alter the E, QF, LF and TPR. The standard drug also significantly increased the E (P < 0.001), QF (P < 0.001), LF (P < 0.001) and TPR (P < 0.001) with respect to the control animals (Table 3).

**Table 3:** Effect of 50% ethanolic extract of *M. fragrans *(nutmeg) on penile reflexes (test for potency) in male rats>

Parameters	Mean Frequency ± SEM				
	
	Control (10 ml/kg)	Nutmeg (100 mg/kg)	Nutmeg (250 mg/kg)	Nutmeg (500 mg/kg)	Sildenafil citrate (5 mg/kg)
Erections (E)	7.67 ± 1.63	7.50 ± 0.42 NS	8.00 ± 0.36*	12.66 ± 0.75***	19.00 ± 2.64***
Quick Flips (QF)	5.17 ± 0.75	5.50 ± 0.49 NS	5.83 ± 0.56 NS	8.66 ± 0.36***	17.30 ± 4.13***
Long Flips (LF)	2.17 ± 1.17	3.33 ± 0.30 NS	4.50 ± 0.42**	8.50 ± 0.42***	12.00 ± 2.26***
Total Penile Reflexes (TPR)	15.01 ± 3.55	16.33 ± 1.21 NS	18.33 ± 1.34*	29.82 ± 1.53***	48.30 ± 9.03***

Tabular values are mean ± SEM, n = 6 (number of animals in each group); significant difference from control, NS: Not significant. *P < 0.05, **P < 0.01; ***P < 0.001.

### Adverse effects

No treatment-related overt signs of toxicity, stress and changes in behaviour were observed. The food and water intake of all the treated animals remained similar to those of the control group.

### Acute toxicity studies

No mortality and changes in the behaviour were observed in all the treated and control groups of mice up to a dose of 4000 mg/kg.

## Discussion

The present study was aimed to investigate the aphrodisiac effect of nutmeg extract (50% ethanolic) along with its acute toxicity using various animal models. The study exhibits a marked change in sexual behaviour of male rats. The results of the present investigations show that the test drug significantly increased the Mounting Frequency (MF) and Intromission Frequency (IF) as compared to control group. However, the standard drug produced a greater increase in thse parameters. The MF and IF are considered the indices of both libido and potency. Thus, the increase in the MF and IF, indicates that nutmeg, along with increasing libido, probably also increases the potency. The significant increase in the Ejaculatory Latency (EL) suggests that the extract and standard drug prolonged the duration of coitus. The significant increase in the EL in both first and second series as well as the decrease in the Post Ejaculatory Interval (PEI), i.e. the refractory period between first and second series of mating, suggest that the test drug intensified sexual activity in a sustained manner. The test drug also caused a significant reduction in the Mounting Latency (ML) and Intromission Latency (IL) as compared to control animals, while a highly significant decrease was observed in the ML of animals treated with the referent drug. This also provides an evidence for aphrodisiac effect of the test drug. These findings show that the test drug produces a striking enhancement of over-all sexual performance of normal animals.

MF after penile anesthetization of rats is a reliable index of 'pure' libido and the penile reflexes of the rats are a good model of 'pure' potency [29]. Therefore, in the present study the extract was also studied for effect on these components of sexual behaviour.

The effect of the test drug on libido was studied by assessing the MF after genital anaesthetization which does away with the reinforcing effect of genital sensation thus affording the study of pure libido or intrinsic sexual desire. During the experiment the test drug produced a significant increase in the MF of sexually normal male rats. Whereas, the MF was much reduced in control, test and standard animals in comparison with the MF of corresponding groups in mating behaviour test where the penis had not been anaesthetized. However, the test for libido revealed that Intromission and Ejaculation were absent in all groups of animals, as the genital sensations which are absent due to penile anaesthetization are necessary for the development of these two events. Thus, it may be inferred that the test drug produced a striking increase in 'pure' libido.

The test for potency exhibited that the extract significantly increased the frequency of all the components of penile reflexes: Erections (E), Quick Flips (QF) and Long Flips (LF) as compared to control group, but comparatively less than the standard drug. The aggregate of these penile reflexes (TPR) was also significantly increased in both test and standard animals. This indicates that the test drug increases 'pure'potency also.

Although the effect of the extract on 'pure' libido and 'pure' potency was evaluated by using two different methods, a rough comparison of the results indicates that the test drug augmented both libido and to an equal extent potency. The positive inferences from the specific tests for libido and potency substantiate the indications of the mating behaviour test to show in a rather conclusive manner that the test drug enhances both the libido and potency in normal male animals. These conclusions are further supported by an earlier study reporting libido and potency increasing effect of nutmeg in mice[23].

In addition, nutmeg, a well know spice and a herbal drug is widely used in Unani medicine without any known or recorded toxicity in the management of male sexual disorders. Such herbal drugs may be directly used, without any toxicity testing. However, when an extract or active fraction of such drug is used it is better to evaluate possible toxicity. Although it is the normal practice to determine the LD_50 _value, now it is acceptable to limit the study to an acute toxicity test using multiple doses including reasonably high doses of the drug [32].

In this connection the test drug was also subjected to an acute toxicity testing and it was tested up to a high concentration of 4000 mg/kg, orally (eight times more than the aphrodisiac dose, evaluated in the present study). Even at this dose the extract did not produce signs of toxicity or treatment- related adverse effects in the tests for aphrodisiac activity. This suggests that its short term use for this purpose is apparently safe.

With regard to the mechanism of the test drug, it is difficult to explain the exact mechanism responsible for improving sexual function. The drug induced changes in neurotransmitter level or their action at cellular level could change sexual behaviour [33]. Nutmeg is mentioned in ethnomedical literature as nervous stimulant [8]. The extract (50% ethanolic) of nutmeg is also reported to have nervous stimulant action in male albino rats [34]. Thus, the resultant aphrodisiac activity of the test drug might be attributed to its nervous stimulating property. Preliminary phytochemical studies indicate the presence of sterols, phenols, alkaloids and amino acids in the extract. Hence, the sexual function improving effect of the test drug might be due to the presence of such compounds. Moreover, nutmeg, merits further studies for detailed sexual function improving activities, especially at higher doses. In addition, further research is also needed for the identification of its active constituent (s) responsible for sexual function improving activities and the mechanism by which it augments sexual function.

## Conclusion

The resultant significant and sustained increase in the sexual activity of male rats, with out any conspicuous adverse effects and toxicity, suggests that nutmeg possesses clinically applicable aphrodisiac activity, and also lends support to the claims for its traditional usage as sexual function enhancing medicine. Further, the study also indicates that the aphrodisiac effects of the test drug may be due to its nervous stimulating property. Thus, it may prove to be an effective and safe alternative remedy in sexual disorders.

## Competing interests

The author(s) declare that they have no competing interests.

## Authors' contributions

T-Supervised the design and coordination of the study.

SA- Practically conducted the design of the study.

AL- Participated and performed the statistical analysis.

IA- Participated and performed the statistical analysis.

KA-Supervised the design and coordination of the study.

All authors read and approved the final manuscript.

## Pre-publication history

The pre-publication history for this paper can be accessed here:


